# Decision support for hospital bed management using adaptable individual length of stay estimations and shared resources

**DOI:** 10.1186/1472-6947-13-3

**Published:** 2013-01-07

**Authors:** Robert Schmidt, Sandra Geisler, Cord Spreckelsen

**Affiliations:** 1 Institute for Medical Informatics, RWTH Aachen University, Pauwelsstr. 30, Aachen, 52074, Germany; 2 Chair of Computer Science 5 - Information Systems, RWTH Aachen University, Ahornstr. 55, Aachen, 52056, Germany

## Abstract

**Background:**

Elective patient admission and assignment planning is an important task of the strategic and operational management of a hospital and early on became a central topic of clinical operations research. The management of hospital beds is an important subtask. Various approaches have been proposed, involving the computation of efficient assignments with regard to the patients’ condition, the necessity of the treatment, and the patients’ preferences. However, these approaches are mostly based on static, unadaptable estimates of the length of stay and, thus, do not take into account the uncertainty of the patient’s recovery. Furthermore, the effect of aggregated bed capacities have not been investigated in this context. Computer supported bed management, combining an adaptable length of stay estimation with the treatment of shared resources (aggregated bed capacities) has not yet been sufficiently investigated. The aim of our work is: 1) to define a cost function for patient admission taking into account adaptable length of stay estimations and aggregated resources, 2) to define a mathematical program formally modeling the assignment problem and an architecture for decision support, 3) to investigate four algorithmic methodologies addressing the assignment problem and one base-line approach, and 4) to evaluate these methodologies w.r.t. cost outcome, performance, and dismissal ratio.

**Methods:**

The expected free ward capacity is calculated based on individual length of stay estimates, introducing Bernoulli distributed random variables for the ward occupation states and approximating the probability densities. The assignment problem is represented as a binary integer program. Four strategies for solving the problem are applied and compared: an exact approach, using the mixed integer programming solver SCIP; and three heuristic strategies, namely the longest expected processing time, the shortest expected processing time, and random choice. A baseline approach serves to compare these optimization strategies with a simple model of the status quo. All the approaches are evaluated by a realistic discrete event simulation: the outcomes are the ratio of successful assignments and dismissals, the computation time, and the model’s cost factors.

**Results:**

A discrete event simulation of 226,000 cases shows a reduction of the dismissal rate compared to the baseline by more than 30 percentage points (from a mean dismissal ratio of 74.7% to 40.06% comparing the status quo with the optimization strategies). Each of the optimization strategies leads to an improved assignment. The exact approach has only a marginal advantage over the heuristic strategies in the model’s cost factors (≤3*%*). Moreover,this marginal advantage was only achieved at the price of a computational time fifty times that of the heuristic models (an average computing time of 141 s using the exact method, vs. 2.6 s for the heuristic strategy).

**Conclusions:**

In terms of its performance and the quality of its solution, the heuristic strategy RAND is the preferred method for bed assignment in the case of shared resources. Future research is needed to investigate whether an equally marked improvement can be achieved in a large scale clinical application study, ideally one comprising all the departments involved in admission and assignment planning.

## Background

Bed capacity is a crucial but limited hospital resource. Therefore, professional bed management aims at an optimal allocation of beds, one involving short waiting periods for the patients and a low rate of canceled admissions, yet with a high occupancy rate. Optimal allocation is hampered by the inherent uncertainty of the patients’ actual length of stay. Furthermore, the bed capacity of a ward tends to be increasingly shared by different clinical units. Bed management is often part of the more general effort at improving patient treatment and maintaining a constant throughput of patients. Currently, many hospitals have formed teams of case and bed managers dedicated to these tasks [1,2].

### Bed management—the current situation in German hospitals

Since 2003, drastic organizational changes have taken place in German hospitals. These were triggered by the ending of payments to the hospital for individual treatments and instead a lump sum compensation based on the internationally established classification of Diagnosis Related Groups (DRG) [3] has been introduced. The current compensation scheme calculates the costs of treatment based on the average length of stay (LoS) of a patient according to the assigned DRG. Therefore, a marked economic loss for the hospital could be the result if the actual LoS exceeds the average. Furthermore, only the number of actually treated cases matters, solely providing the clinical infrastructure is no longer rewarded. Due to these changes, the traditional one-to-one link between a ward and a specific clinic was abandoned. Today, patients treated by different clinical units may be assigned to the same ward, provided that there are no medical reasons not to do so. The rate of elective patients varies between 30% and 80%, depending on the clinical unit [4]. Up to 80% of a surgical unit’s patients are elective, and their admission could thus be planned in advance. Patient admission planning and assignment in German hospitals are often performed by the Case Management and Standard Care departments. The patient admission planning decision process of case managers has been reported to be a complex process, heavily influenced by a multitude of factors [5].

In the course of this work, a comprehensive requirements analysis based on five semi-structured interviews with several representatives of the Standard Care (SC), Case Management (CM), and IT departments of the local hospital RWTH University Hospital Aachen, Germany (UK Aachen) has been performed. The CM department has been established at the hospital to improve all processes concerning the treatment, nursing, and after-treatment of the patients. Preliminary investigations revealed an incremental shift of the tasks of patient admission planning, from clinics to case managers. Thus, the CM department could be regarded as a centralized unit, responsible for admission planning. The SC department was established as an interdisciplinary department responsible for nursing the patients who are treated in different clinics. The SC department is currently facing manifold challenges concerning bed management, e.g., assigning patients so that the spatial distance to the treating clinic is minimized. The IT department has to provide all necessary data for resource planning and an IT infrastructure for decision support.

The interviewees of the SC and CM departments are primarily involved in bed and case management at UK Aachen. All interviews were prepared in advance, recorded, and analyzed afterwards. The interviews lasted approximately 30 to 90 minutes each. The interview protocols were transcribed immediately after the interviews. The interviews aimed at: 

1. The elicitation of information about the departments, the responsibilities, and the daily challenges of the persons in charge concerning admission planning and assignment.

2. The identification of those aspects that must be respected in the development of the Decision Support System (DSS).

#### Results of the interviews

According to the interviews, bed management has gained increasing attention in Germany since the establishment of the new financial compensation model for hospitals (based on Diagnosis Related Groups). There is a great need for computer-based decision support in this context, due to the high complexity of the planning problem.

Bed management aims at finding a suitable admission date with respect to the preferences of the patient and the bed capacities available. The increasing responsibility of the hospital’s case managers for the patient admission planning tasks has been reported to have resulted in a more centralized planning process and in reducing the inherent organizational complexity of a decentralized planning scenario. Current approaches to bed management using multi-agent systems [6-8] involve decentralized planning processes and thus were not further considered. Futhermore, the interviewees reported an inherent uncertainty in the LoS and in the duration of the specific treatment steps, which results in uncertainty of the bed capacity available at a given time. Only a fraction of the admissions are planned in advance. Acute patients must be assigned immediately. The possible planning time frame of a patient depends on the individual case of the patient. It was suggested by the interviewees that patients may be categorized into priority classes, taking their individual treatment needs into account. The grouping of patients with respect to their treatment needs has recently been considered by Wang et al. [9] as well, who categorize patients into different priority classes with respect to their state of illness. The interviewees reported that the planning time frame can be between a day and several months. In general, patients are scheduled within two months. In practice, patients are categorized into priority groups with respect to their planning time frame. Three priority groups have been depicted by the interviewees: priority 1 patients must be scheduled within 24 hours, priority 2 patients within one week, and priority 3 patients may be scheduled fairly long-term. Restrictions exist even for admissions which can be planned in advance: e.g., treatments may have to be started before or after a given date, for medical or personal reasons. Additional restrictions involve the preferred, allowed, or excluded combinations of patients in the same room or a patient’s demand for a bed in a single room. Furthermore, an increasing relaxation of the former tight linkage between clinics and wards has been reported. Interdisciplinary units, such as the Standard Care department, were established with the main objective of nursing patients treated by different clinics. However, clinics may prefer special wards for their patients for medical or organizational reasons. Lists are used for structuring the planning task. The overall planning collective on a list does not exceed 100 patients in general. Further aspects and statements from the interviews are summarized in Table 1.

**Table 1:** Summary of aspects and statements from the requirements interviews

**Aspect**	**Statement**
Are patient admissions plannable?	The majority of admissions are plannable, although with different deadlines.
How do you perform the planning task?	We use lists for planning and categorize patients into three priority groups.
What are the characteristics of the priority groups?	The priority is mainly characterized by the length of the planning period.Admission within: Priority 1 24 hours Priority 2 a week Priority 3 long term, in general within two months
How many patients are on a list?	Up to 100.
What planning aspects do you consider?	We consider: •Treatment priority •Dependencies with respect to the patient’s treatment process •Availability of the required resources •Patient’s preferences with respect to the patient’s condition and health insurance •Uniform resource utilization
How do you use the clinical IT system?	We use the clinical information system for: •Reviewing resources •Communication •Booking of resources
Is decision support provided by IT?	No, not directly. Indirect support is provided by outlining the planned resources.
What are your demands on a DSS?	Modifications of the LoS, treatment, and care plans must be considered as early as possible. Furthermore, the DSS must pay regard to: •Patient’s treatment priority •Patient’s preferences •Utilization of the wards •Provision and utilization of emergency beds •Easy and interactive usage, allowing user modifications and providing re-computation Dependencies within the patient’s treatment process shall be considered early. Furthermore, there shall be supported: •Periodically occurring treatments •Patient preferences shall be modifiable

The new challenges concerning bed management in German hospitals can be summarized as follows: 

1. Wards must be considered to be partially shared and central resources.

2. Patients treated by different clinical units may be assigned to the same ward.

3. There are special limitations due to medical, insurance, or social reasons, which restrict the sharing of the same room or even ward by different patients (e.g., the exclusion of mixed gender rooms).

4. The planning process has to cope with the inherent uncertainty of the outcome of the single treatment steps and the overall duration of the patient’s stay.

5. Patients may be planned in advanced and categorized into priority groups reflecting the urgency of the treatment.

### Related work

The challenges of bed management without computer assistance leading to special training programs for the persons in charge have been analyzed intensively by Proudlove et al. [10,11]. Bed management has to solve optimization problems in a context with a high level of uncertainty: the outcome of a treatment cannot be predicted fully, and emergency patients need to be treated immediately.

Case Management as described above has been reported to potentially improve the treatment and care trajectory of cancer patients and is increasingly being implemented in the organizational structure of hospitals [12]. The challenges for case managers are more complex than those for bed managers in terms of resource allocation planning, since case managers need to consider the entire clinical pathway. Due to interdependencies between resources, the decision making process concerning resource allocation was reported to be highly sophisticated [5]. Besides the complex interdependencies, the variability in the usage of resources plays an important role [13]. Gallivan et al. investigate the variability of the patients’ LoS in intensive care after cardiac surgery [13]. Their results indicate that this variability has a considerable impact on the intensive care capacity requirements. They conclude that a booking admission system should be treated with caution regarding inpatient admissions if there is a high variability in the LoS.

Mathematical approaches and computer-based assistance designed to solve the optimization tasks described above were early on proposed and developed. Queuing Theory and Compartmental Flow Models have been successfully applied in a clinical context: McClean reports on a Decision Support System (DSS) based on a compartmental flow model [14]. Fomundam and Herrmann give an overview of the application of queuing theory approaches in the health care sector [15]. A recent review of queuing models applied to clinical problems especially addresses patient planning approaches [16]. Approaches based on queuing theory have also been frequently combined with simulation models. For example, Cochran and Bharti describe a multistage methodology to balance inpatient bed utilization in a hospital [17]. The approach combines a queuing model and a discrete event simulation in order to achieve flow-maximization in the system. However, queuing theory approaches are unsuitable for providing decision support on the operational level in the presence of frequent revisions of LoS estimates, since the inherently highly variable dynamics resulting from the revisions would not be sufficiently taken into account. In the related field of operation room planning, Discrete Event Simulation has been applied to situations where queuing models cannot be used accurately [18].

Stochastic scheduling approaches have already been proposed for admission planning tasks as well. Connors describes a stochastic scheduling algorithm employing deterministic and stochastic constraints [19]. The patient’s characteristics and requirements as well as the hospital’s status are considered by the algorithm. The patient’s LoS is modeled stochastically, reflecting the probability of the LoS. A gamma density function is used to model the patient’s LoS. The aggregation of similar resources with predefined characteristics is not considered. The LoS estimates cannot be modified after the patient is admitted and are thus static.

Stochastic scheduling has been applied to the task of maximizing operating room utilization, which bears considerable similarity to the bed assignment problem: Arnaout developed and evaluated an approach [20] based on the *Longest Expected Processing Time first* (LEPT) strategy [21]. The LEPT strategy is a stochastic scheduling strategy that processes jobs in decreasing order with respect to the expected processing time. Similarly, the *Shortest Expected Processing Time* (SEPT) strategy processes jobs in increasing order. Both strategies are stochastic extensions of the deterministic strategies, *Longest Processing Time* (LPT) and *Shortest Processing Time* (SPT). Variants of the LEPT and LPT strategies have been applied in the context of surgery planning as well [22-24]. Hans et al. consider a robust surgery loading problem with the subject of surgery assignment to operating room day schedules [22]. A part of their algorithm contains a dispatching rule which is based on the LEPT strategy. The LPT and SPT strategies are considered by Lamiri et al. as a constructive heuristic in order to calculate a basic solution of the planning problem [23]. The LEPT and SEPT strategies are used to compute an approximate solution of the mathematical model within this work as well.

Mathematical Programming approaches have also been successfully applied to health care related problems: Zhang et al. used a Mixed Integer Program (MIP) to optimize the capacity allocation of operating rooms to specialties [25]. MIPs and Binary Integer Programs (BIPs) belong to the complexity class of NP-hard problems and are thus in general challenging [26]. Several approximation and heuristic strategies have been developed to compute a solution in reasonable time, which, however, cannot be guaranteed to be optimal [27-30]. Heuristic approaches differ from approximate ones in that they include an unforeseeable error in the approximate solution. Hans et al. describe optimization models for surgery planning: their approach tries to maximize the capacity utilization, minimize the risk of overtime, and minimize the number of canceled admissions [22,31]. That approach allows operating room capacity to be freed up for additional surgeries. Lamiri et al. propose several approaches to improve the scheduling of surgeries [23,32,33]. Surgery times as well as operating room capacities were modeled by random variables in order to represent their inherent stochastic variability. Belien et al. proposed and evaluated models for generating surgery schedules with leveled resulting bed occupancy [34]. However, the existing approaches in general focus on the rooms or clinical staff. Hospital beds and ward capacities are usually regarded as an auxiliary condition—if considered at all.

Chow et al. describe a combination of a mixed integer optimization model and a Monte Carlo simulation method in order to improve scheduling practices of operating room use and the resulting downstream bed utilization [35]. Surgical schedules are simulated with reference to historical case records and the resulting bed requirements are predicted accordingly. The mixed integer optimization model allows scheduling both surgeon blocks and patient types. The simulation allows predicting the impact of the scheduling rules.

Demeester describes a patient admission scheduling algorithm considering hospital beds and supporting operational decisions concerning hospital bed assignments [36]. That approach assigns patients to individual rooms and hospital beds for exact dates. The hospital beds and hospital rooms are considered a single, individual resource and it does not contemplate a suitable variety of hospital beds and rooms in a ward.

### Aim

This paper addresses the feasibility of a computer-supported bed management taking into account aggregated bed capacities (shared resources) and LoS estimates that can be individually updated during the patients’ treatment. This study aims at answering the question of how to choose and combine modeling and optimization approaches in order to fulfill the following requirements (which were identified by the preliminary investigation): 

1. The treatment priority of the patients must be the major concern and must, thus, be considered with rights to appeal.

2. The approach should address the bed capacity of a ward by aggregating beds into groups, instead of focusing on the assignment of patients to individual beds. In these groups, beds are equivalent with respect to the limitations mentioned above.

3. The planning process should be based on LoS estimates and representational means of uncertainty, namely appropriate probability distributions for the LoS adjusted to the variance of the empirical data.

4. It should be possible to dynamically adapt a plan to changing estimates.

The approach to be presented in this paper aims at improving the efficiency and effectiveness of the bed and case management process. This approach, fulfilling the above criteria, should enable a decision process that respects the patients’ treatment priorities and individual preferences (e.g., gender, private or double room, diagnosis), relies on ward capacities, deals with the inherent uncertainty of the patients’ recoveries, and allows a dynamic adjustment to changing LoS estimates. Due to the complexity of this optimization task, different types of algorithmic approaches (e.g., exact solution vs. heuristic strategies) needed to be evaluated with respect to their performance and the quality of their results.

As far as we know, none of the published approaches fulfills all these requirements.

## Methods

### Rationale of the approach

As found by the requirements analysis, the LoS is an important factor in estimating the capacity utilization. Therefore, an adequate decision support should rely on a system architecture which supports an interactive adjustment of the estimated LoS by the users. The adjustment of the LoS estimates yields a more precise estimate of the ward’s usage rate, since the usage rate can be calculated using the up to date LoS estimates. Our approach focuses on the assignment of patients to bed contingents respecting the given patient preferences, in contrast to an assignment of patients to individual beds as carried out, for instance, by Demeester et al. [36]. The LoS is treated as a stochastic variable, hence, optimization has to be based on expected values. Different optimization strategies will be compared, including the exact solution and three heuristic approaches.

### Software architecture

**Figure 1:**
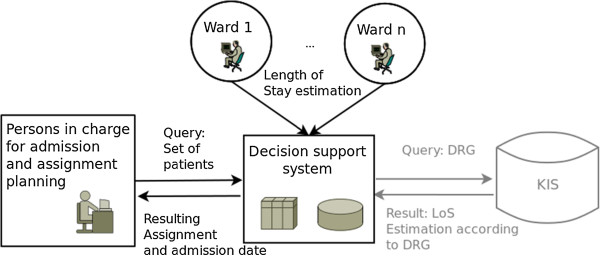
**Architecture of the DSS.** The architecture of the DSS consists of two interfaces: one for LoS submission and another one for query submission. The LoS estimate can be submitted at any time and is immediately considered in the decision support. An initial LoS estimate may be derived from the clinical information system (KIS). An admission planning query can be submitted at any time as well. The DSS calculates an optimal admission date and assignment for the patient group.

**Figure 2:**
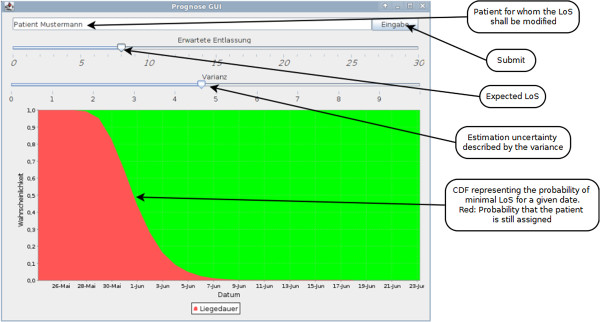
**Length of stay submission interface.** The expected LoS as well as an uncertainty factor may be entered. Visual feedback is provided by presenting the resulting cumulative distribution function (CDF) and highlighting the assignment probability in red.

Figure 1 shows the software architecture of the DSS developed. The ward staff in charge of a patient may submit a patient LoS estimate at any time. An initial LoS estimate may be ascertained using data from the clinical information system (gray). A special user interface, Figure 2, supports the LoS estimate submission with an interactive diagram.

**Figure 3:**
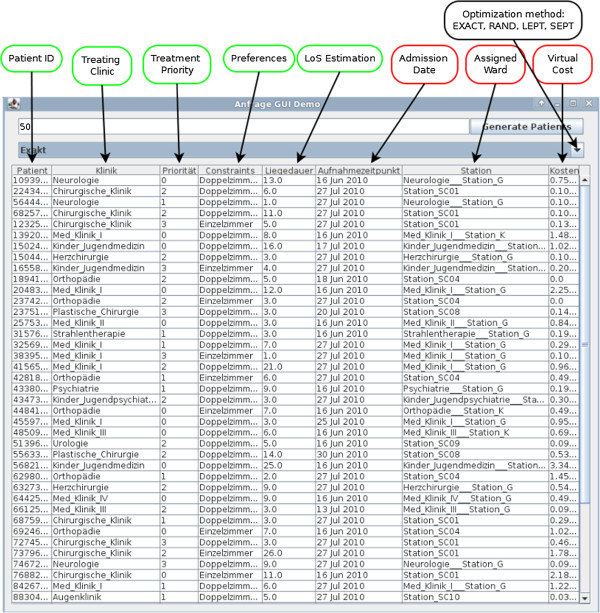
**Query interface for admission and assignment planning.** Each Row contains a patient dataset. The columns labeled with green contain the data that has to be entered in advance. The red labeled columns contain the result set of the DSS. The dropdown menu allows choosing the optimization strategy (EXACT, RAND, LEPT, SEPT) to be used.

The persons in charge of admission planning and assignments may submit queries to the system. Such queries specify a set of patients, whose admission dates and assignments have to be planned. The decision support module calculates an optimal admission date and assignment for each patient. The implemented prototype user interface is shown in Figure 3.

### Optimization model

#### Length of stay estimation

The LoS of patient *i* is modeled by a log-normally distributed random variable *D*_*i*_, representing the LoS estimate. The density *P*(*D*_*i*_ = *t*) represents the probability that patient *i* is assigned to a ward at time *t*. The cumulative distribution *P*(*D*_*i*_≥*t*) = 1−*P*(*D*_*i*_ <*t*) = *p*_*it*_ represents the probability that patient *i* is assigned to a ward at least until time *t*. This event can be regarded as a Bernoulli trial with success probability *p*_*it*_. In general, the distribution may be chosen arbitrarily. Nonetheless, Marazzi et al. [37] and Ruffieux et al. [38] showed that the log-normal distribution is superior to other closed-form distributions in the context of LoS modeling.

The persons in charge submit their estimates of the expected date of discharge and specify their uncertainty by defining a time interval for the patient’s discharge. The system interprets the submitted parameters as the expectation value *μ* and the variance *σ* of the log-normal distribution.

#### Expected ward capacity

The expected free capacity of a ward at a given time is calculated based on the LoS estimates of the patients assigned to the ward in question within the time frame being considered. The expected free capacity can be updated dynamically based on revised LoS estimates.

Let *j* be the ward in question with a total capacity of *n*_*j*_ beds, and let *m*_*j*_ be the number of patients assigned to the ward. In order to derive the probability distribution of the ward’s usage rate, a Bernoulli distributed random variable *X*_*it*_ is introduced. The event *X*_*it*_ = 1 represents the event that patient *i* is still assigned to the ward in question at time *t* with the corresponding probability *P*(*X*_*it*_ = 1) = *p*_*it*_. The probability distribution of the use of ward *j* at time *t* can now be calculated with the random variable $S_{\text{jt}}=\overset{m_{j}}{\underset{i=1}{\sum }}X_{\text{it}}$. The densities *P*(*S*_*jt*_ = *k*), *k* = 0,…,*p*_*j*_ may be approximated through a reduction to the normal distribution, according to the central limit theorem. However, the expected use *E*[*S*_*jt*_] of the ward *j* at time *t* can be calculated directly, since $E[S_{\text{jt}}]=E[\overset{m_{j}}{\underset{i=1}{\sum }}X_{\text{it}}]=\overset{m_{j}}{\underset{i=1}{\sum }}E[X_{\text{it}}]=\overset{m_{j}}{\underset{i=1}{\sum }}p_{\text{it}}$. The expected free capacity is, thus, *n*_*j*_−*E*[*S*_*jt*_]. The expected usage rate is $\frac{E[S_{\text{jt}}]}{n_{j}}=:c_{\text{jt}}$.

#### Affinity between clinics and wards

A patient cannot be assigned to an arbitrary ward in the hospital. In general, only a subset of the wards is suitable for a given patient. This subset depends on the clinic responsible for the treatment of the patient. Furthermore, usually there exists a ranking between suitable wards. The suitability (and ranking) of wards with respect to a clinic is modeled by a corresponding mapping (affinity), assigning a value *α*∈[0,1] to each pair of a ward and a clinic.

#### Cost factors

Aspects of an assignment benefit are represented by cost factors: *affinity costs*, *ward occupancy*, *change of ward occupancy*, and *assignment delay*. Affinity costs: The affinity *α*∈[0,1] introduced above represents a cost factor which has to be defined by the administrator of the DSS for each pair of ward and clinic in advance. A high affinity (close to 1) represents a high preference of the ward for nursing a patient treated in the corresponding clinic. If *α*=0, patients treated in the corresponding clinic cannot be assigned to the ward in question. This cost factor is referred to as the *α* cost factor. Ward occupancy: As solely providing a clinical infrastructure is no longer rewarded by the DRG-based compensation scheme, the use of the ward’s capacity is an important factor in the hospital’s economic status. Wards should have a high average rate of use. Therefore, the optimization approach needs to consider the estimated ward usage as a cost factor (which will be referred to as the *β* cost factor). The *β* cost factor is defined for each patient *i*, ward *j*, and admission date *t*: $\overset{E[D_{i}]}{\underset{m=t}{\sum }}c_{\text{jm}}$.

Change of ward occupancy: Frequent changes of the ward during a clinical stay exposes the patients and the nursing staff to additional stress and should therefore be avoided. The corresponding measure is referred to as the *γ* cost factor, and is defined for patient *i*, ward *j*, and admission date *t* by $\overset{E[D_{i}]}{\underset{m=t}{\sum }}|c_{j(m+1)}-c_{\text{jm}}|$.

Assignment delay: The *δ* cost factor weights the delay until the patient’s assignment by their treatment priority in order to reduce the waiting time and to provide a timely start for urgent treatments.

### Mathematical program

The formal description of the admission planning and assignment problem is given by a Binary Integer Program (BIP) [27].

#### Parameters

*b*=1,…,x=|ℬ| index of patient preferences *i*=1,…,n=|P| index of patients *j*=1,…,m=|S| index of wards *t*=1,…,k=|T| index of allowed days of admissions *U*_*jtb*_ random variable representing the number of used beds of ward *j*, fulfilling the patient preference *b* at date *tV*_*i*_ random variable representing the LoS of patient *iE*(*U*_*jtb*_) expected number of used beds on ward *j* at date *t*, fulfilling the patient preference *bE*(*V*_*i*_) expected LoS of patient *iK*_*jb*_ overall bed capacity of ward *j*, fulfilling the patient preference *b*$c_{\text{jtb}}=\frac{E(U_{\text{jtb}})}{K_{\text{jb}}}$ cost resulting from an assignment to ward *j* at date *t* by a patient with preferences *bC*_*i*_ treating clinic of patient *im*_*α*_ affinity weight factor *m*_*β*_ ward usage weight factor *m*_*γ*_ ward usage change weight factor *m*_*δ*_ admission delay weight factor

#### Mappings

• AFF:S×K→[0,1], Mapping of the affinities between wards and clinics

• Cons:P×ℬ→P, Mapping of patients who demand the preference b∈ℬ

• Cons:S×ℬ→S, Mapping of wards satisfying the patient preference b∈ℬ

• $L_{\text{max}}:P\to T$ Mapping of the maximal LoS of a patient within the set P

• Prio:P→{0,1,2,3}, Mapping of patients to their treatment priority

#### Decision variable

•

$$x_{\text{ijt}}=\left\{\begin{array}{ll} 1, & \;\text{iff patient i is admitted to ward j at time t}\\ 0, & \;\text{else} \end{array}\right)$$

#### Objective function

(1) $$\text{min}\left\{\underset{b\in ℬ}{\sum }\underset{i\in \text{Cons}(P,b)}{\sum }\underset{j\in \text{Cons}(S,b)}{\sum }\underset{t\in T}{\sum }x_{\text{ijt}}\cdot \right)$$

(2) $$\left(m_{\alpha }\cdot \text{AFF}(C_{i},j)+\right)$$

(3) $$m_{\beta }\cdot \left(\overset{\left\lceil E(V_{i})\right\rceil }{\underset{m=t}{\sum }}c_{j,m,b}\right)+$$

(4) $$m_{\gamma }\cdot \left(\overset{\left\lceil E(V_{i})\right\rceil }{\underset{m=t}{\sum }}|c_{j,(m+1),b}-c_{j,m,b}|\right)+$$

(5) $$\left(\left(m_{\delta }\cdot \frac{1}{1+\text{Prio}(i)}\cdot \left(1-\frac{1}{1+\sqrt{t}}\right)\right)\right\}$$

#### Constraints

•

$$\forall b\in ℬ:(\underset{i\in \text{Cons}(P,b)}{\sum }\underset{j\in S}{\sum }\underset{t\in T}{\sum }x_{\text{ijt}}=|\text{Cons}(P,b)|)$$

•The above restriction represents the requirement that an admission date and a ward shall be derived for every patient. The set of patients is partitioned according to the patient preferences.

•In order to ensure the solvability of the BIP, a dummy ward with high capacity has been modeled. Patient’s assignment to the dummy ward is accompanied by an enormous penalty cost and is interpreted as a dismissal.

•

$$\forall b\in ℬ:(\forall i\in \text{Cons}(P,b):(\underset{j\in S}{\sum }\underset{t\in T}{\sum }x_{\text{ijt}}\leq 1))$$

•The above restriction implements the requirement that no more than one admission date and one ward is calculated for a given patient, thus preventing multiple assignments.

•

∀b∈ℬ:(∀j∈Cons(S,b):(∀t∈T:

$$\left(\underset{i\in \text{Cons}(P,b)}{\sum }\overset{t}{\underset{t_{1}=\text{max}(0,t-L_{\text{max}})}{\sum }}x_{\text{ij}t_{1}}\leq K_{\text{jb}}-E(U_{\text{jtb}}))\right)$$

The capacity of a ward shall never be exceeded. To prevent exceeding the capacity of the ward, all assignments of previous admission dates must be considered. The expression *K*_*jb*_ − *E*(*U*_*jtb*_) represents the expected number of free beds that fulfill the patient’s preference *b* at date *t* of ward *j*. Exceeding the ward’s bed capacity can find its cause in the following scenarios: 

1. Earlier patient assignments to ward *j* have led to the excess the ward’s capacity.

2. The current patient assignment exceeds the ward’s capacity.

To prevent both cases of incorrect assignment, a time-window is defined. The time-window is defined by the expression of the inner sum: $\overset{t}{\underset{t_{1}=\text{max}(0,t-L_{\text{max}})}{\sum }}$.

 The variable *L*_*max*_ represents the maximum LoS of the considered set of patients.

The BIP is tackled by the software tool SCIP [39] within the EXACT approach.

### Optimization methods

The admission planning and assignment problem has been described as a BIP in the previous section.

#### Exact solution

The exact solution represents the mathematically exact and optimal solution of the BIP. Several software tools have been developed to provide methods to solve such problems.

The software tool SCIP^a^[39], currently the most powerful non-commercial software tool to solve BIPs^b^, is used in this project. The exact approach to solve the program is referred to as the EXACT approach. Notably, dismissals are modeled as assignments of patients to a residual category of beds (at a maximal cost) in order to guarantee the solvability of the assignment problem. Due to the complexity of the exact algorithm, a timeout has to be defined. If the algorithm fails to reach a solution in time, it is stopped without creating an assignment. Thus, a timeout results in dismissals.

#### Heuristic strategies

Several heuristic strategies have been proposed to compute solutions in reasonable time. Pinedo [21] provides an overview of the common heuristic approaches. In heuristic strategies, the assignment problem is usually simplified by a reduction to an online problem instance. An online algorithm tackles a given problem instance in a sequential piece-by-piece manner, whereas an offline algorithm approaches the problem at hand as a whole, considering the relevant interdependencies of its parts [40].

Heuristic strategies involve two parameters: the assignment order of the patients and a cost criterion. The cost criterion represents the individual assignment cost of the current patient, date, and ward.

The assignment order of the patients is determined by the following strategies: Longest Expected Processing Time (LEPT): The LEPT approach sorts the patients in descending order solely according to their expected LoS. Afterwards, it assigns them to the wards sequentially following a greedy minimal cost strategy. Shortest Expected Processing Time (SEPT): The SEPT approach is similar to the LEPT strategy. The only difference is that patients are sorted in ascending order with respect to their expected LoS. Random choice (RAND): The patients to be assigned are randomly chosen. The RAND strategy has been implemented as a baseline in order to analyze the effect of sorting.

All heuristic strategies use a minimal cost strategy for the final assignment by checking all possible assignments for the patient under consideration and greedily selecting the one with minimal cost.

The worst case complexity of the heuristic approaches is $O(|P|\cdot \text{log}(|P|)+|S|\cdot |T|\cdot L_{\text{max}})$ for LEPT and SEPT (due to the sorting procedure) and $O(|S|\cdot |T|\cdot L_{\text{max}})$ for RAND, where |P| is the number of patients to be assigned, |S| is the number of wards to be considered, |T| is the number of days in the planning time frame, and *L*_*max*_ is the maximal LoS.

#### Status Quo approach

In order to allow a comparison between the aforementioned optimization methods (heuristic strategies and optimal solution) and the status quo proceeding as carried out by the human bed managers, the STATQUO approach was defined. STATQUO neglects those cost factors that are usually neglected by the human decision making as well. Therefore, only the affinity factor (*α* factor) and the ward occupancy (*β* cost factor) are considered. The assignment order of patients corresponds to the RAND strategy.

### Simulation

All of the above specified planning strategies were evaluated and compared in a Discrete Event Simulation (DES) [41] study. A virtual hospital environment containing wards and clinics was designed to represent the system. An event represents a new admission and assignment planning task for a group of patients, referred to as the planning collective, of random size. Each event occurs at a point in time and affects the state of the system: the occupancy rate of the wards.

**Figure 4:**
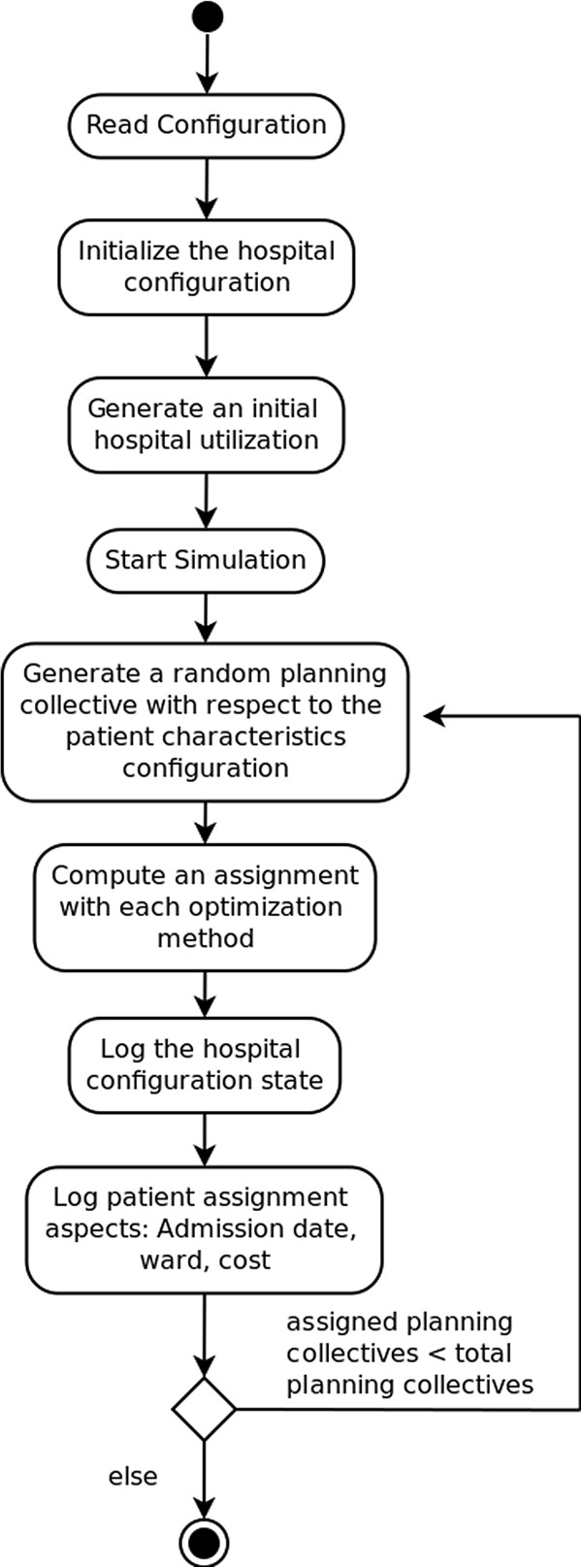
Activity diagram of the simulation flow.

In order to allow a detailed analysis, the states before and after the event are logged as well as the computed assignment of the patients and the computation time. The general proceeding of the simulation is depicted in detail in Figure 4.

#### Simulation environment

A highly configurable simulation environment was developed, allowing a close to reality simulation, based on a realistic hospital model. In each run of the simulation, a group of patients with random configurations is generated, and then at the point of each admission, an assignment strategy was applied to this group. Characteristics such as the detailed assignment costs, the wards’ states before and after the assignment, and the computation time, were logged for further analysis. A simulation is specified in general by the following four constants: 1) PPY: the total number of patients to be considered over one year (the basic time interval of a simulation run), 2) MXP: the maximal number of patients to be assigned to beds in one single assignment cycle (the actual number is randomly chosen from the interval between zero and this maximum), 3) CPD: The number of assignment cycles per day 4) SPF: the shift of the planning frame, (in days), when all CPD assignment cycles have been accomplished.

#### Hospital simulation model

The modeling framework allows to specify a hospital by defining the available clinical units and wards. Different bed capacities can be set up for each ward by giving the number of available beds with specific features (e.g., single vs. double room bed). For each pair of ward and clinical unit, the affinity quantifies whether (i.e., to what degree) it makes sense to assign a patient treated in the clinical unit to a bed of the respective ward.

#### Patient simulation model

Furthermore, the modeling environment is able to generate data sets of patient groups with individual patient characteristics: treatment priority, gender, treating clinic, initial LoS estimate, and individual preferences (single vs. double room, etc.). Valid admission dates are derived from the treatment priority. An exemplary data set for one patient would be: (TreatmentPriority=2, Gender=male, TreatingClinic=Urology, LoS=5, Preference=SingleRoom).

**Figure 5:**
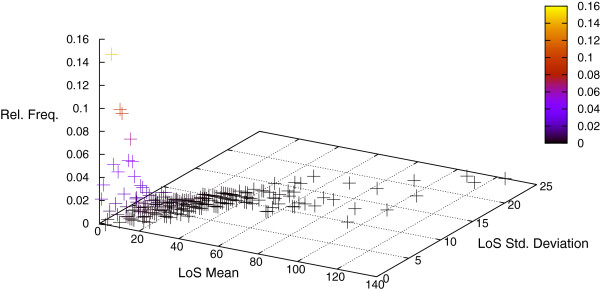
**Mean and variance of the LoS frequency distribution.** Three-dimensional representation of the relative frequency distribution of the mean and variance LoS attributes according to the DRG browser. The relative frequencies are colored to highlight their position.

The individual characteristics of the patients are randomly generated based on given statistical distributions: the LoS distribution is based on publicly available, official data of the German DRG statistics^c^ (LoS mean values, upper and lower LoS bounds for the different groups according to the DRG classification system, and prevalence data). See Figure 5. The statistical distributions of treatment priority, gender, treating clinic, and patient preferences must be defined accordingly in order to perform the simulation.

#### Statistical analysis

The simulation data as well as the simulation results are analyzed by descriptive statistics. The mean, median, standard deviation, first and third quartile are calculated and presented in boxplot diagrams.

#### Ethics approval and informed consent

Neither institutional ethics approval nor written consent from participants were required to perform this study, since all patients were virtually generated according to publicly available statistical data.

Informed consent was provided by the case management and bed management department.

### Hard- and software platform

The DSS was developed and evaluated on a Lenovo Thinkpad R61, Intel Core 2 Duo CPU 2.0 GHz and 2048 MB RAM.

The operating system was Ubuntu 10.04 GNU/Linux i686 running a 2.6.28 Kernel. The major part of the DSS was developed in Java version 6. The software tool SCIP [39] was used to solve the BIP.

## Results

### 

#### Simulation data

The general parameters were set as follows: PPY:= 45,000, MXP:= 110, CPD:= 5, SPF:=1.

#### Hospital environment

The model configuration for this study was derived from the actual situation at UK Aachen using datasets provided by our local medical controllers. Additional information was elicited by interviews with the relevant representatives of the UK Aachen. The model contained 27 clinics, 43 wards of 15 different types, and 72 affinities, quantifying the associative strength between a clinic and a ward based on the results of the interviews within the requirements analysis. The data characterizing the clinics is shown in Table 2 and the data of the wards in Table 3 respectively.

**Table 2:** Case distribution of UK Aachen clinics during the year 2008

**Clinic**	**Inpatient cases**		**Avg. LoS**
	**Absolute**	**Relative Freq.**	
Ophthalmic clinic	2040	4,65%	4,1
Surgical clinic	2704	6,15%	8,9
Gynecological clinic (Breast)	183	0,42%	7,3
Gynecological clinic	88	0,2%	3,8
(Endocrinology)			
Gynecological clinic	2458	5,59%	5,4
Dermatology	1234	2,81%	6,6
Vascular surgery	661	1,5%	9,6
Otolaryngology	2093	4,76%	5,9
Pediatrics	4674	10,63%	5,5
Pediatric psychology	408	0,93%	31,2
Child cardiology	403	0,92%	6,5
Nuclear medicine	237	0,54 %	3,4
Orthopedics	1515	3,45%	9,0
Accident surgery	1160	2,64%	10,1
Palliative medicine	212	0,48%	12,6
Plastic surgery	901	2,05%	11,6
Psychiatry	1693	3,85%	18,4
Radiation therapy	782	1,78%	13,8
Cardiac surgery	1296	2,95%	18,4
Med. Clinic I	7040	16,02%	6,3
Med. Clinic II	1453	3,31%	11,9
Med. Clinic III	3116	7,09%	8,0
Med. Clinic IV	1677	3,82%	7,6
Neurosurgery	1227	2,79%	12,5
Neurology	2697	6,14%	8,6
Urology	1323	3,01%	7,7
Facial surgery	676	1,54%	6,4

Absolute and relative frequencies of inpatient cases and average LoS divided by clinic of UK Aachen in the year 2008.

**Table 3:** Ward types of UK Aachen

			**Beds**	
**Type**	**Overall**	**Single**	**Female Double**	**Male Double**
Ward_G	30	8	10	12
Ward_K	16	2	7	9
Ward_SC01	30	4	11	15
Ward_SC02	32	8	10	14
Ward_SC03	14	2	6	8
Ward_SC04	30	8	10	12
Ward_SC06	32	8	10	14
Ward_SC07	27	6	9	12
Ward_SC08	14	2	6	8
Ward_SC09	32	8	10	14
Ward_SC10	32	8	10	14
Ward_SC11	16	2	7	9
Ward_SC12	32	8	10	14
Ward_SC15	4	2	2	2
DUMMY	1000	300	350	350

Ward types of UK Aachen characterized by the contingent of different bed classes.

#### Patient planning collectives

The LoS distribution was derived from the DRG-Browser^d^ and is portrayed in Figure 5. The distribution of patients treated by specific clinics over the year (treating clinic) was inferred from the annual report of UK Aachen [42] and is summarized in Table 2. The sizes of the planning collective were uniformly chosen from the set {1,…,110} according to the interviews of the requirements analysis. The treatment priority was chosen based on the statistics portrayed in Table 4. The gender distribution was estimated to be 56% male and 44% female patients. The distribution of patient preferences reflected the characteristics of the ward types portrayed in Figure 3, e.g., for “Ward_G”: the probability for a single bed is 8/30 and for a female patient in a double bed room 10/30.

**Table 4:** Distribution of treatment priorities

**Treatment priority**	**Probability**
0	0.1
1	0.2
2	0.3
3	0.4

The treatment priorities and their corresponding statistical occurence rates.

### Results of the simulation

Different assignments were calculated and recorded, for each of the assignment strategies. The results were subsequently analyzed considering the following aspects: 

•The ratio of successful assignments and dismissals

•The performance of the assignment calculation

•The distribution of the cost factors

#### Successful assignments vs. dismissals

**Figure 6:**
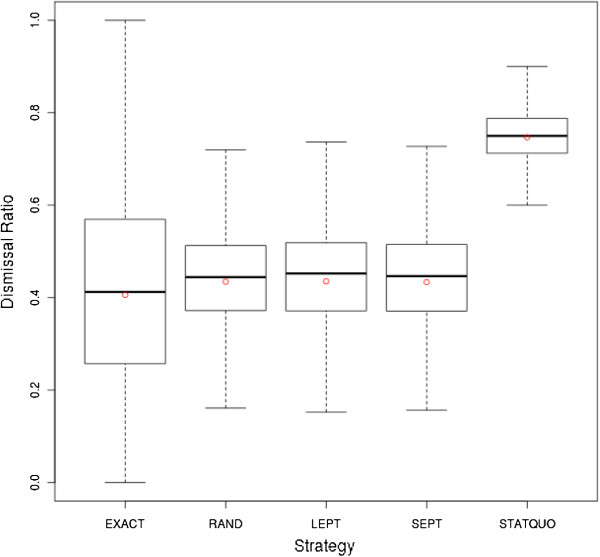
**Boxplot of dismissal ratios refined by strategy.** The Statistical characteristics of the dismissal ratios refined by planning strategy are depicted by a boxplot.

The boxplot in Figure 6 depicts the statistical aspects of the patient dismissal ratio for each planning strategy. The red dot indicates the mean value. The boxplot shows that there is a great similarity in their statistical aspects between the heuristic strategies LEPT, SEPT, and RAND (mean ca. 0.43, median ca. 0.44, first quartile ca. 0.37, third quartile ca. 0.51). Furthermore, the boxplot shows that the EXACT strategy has the lowest mean (0.4060) and median (0.4123) dismissal ratio. However, the range between the lower (0.2571) and upper quartile (0.5696) of about 0.3125 is greater than those of heuristic strategies (ca. 0.14). Thus, the lower mean and median outcome is accompanied by a higher variance (0.0455 for the EXACT and ca. 0.027 for the heuristic strategies). The simple status quo proceeding has a high dismissal ratio, almost 0.7471, and thus discloses the advantage of using one of the proposed admission and assignment strategies.

**Figure 7:**
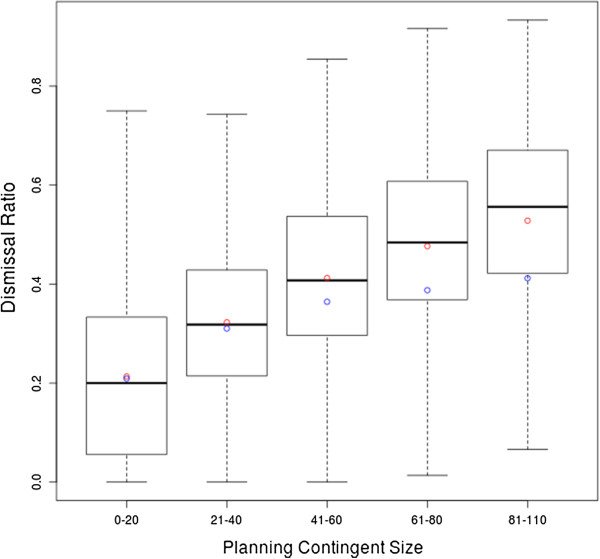
**Boxplot of the EXACT strategy’s dismissal ratios refined by planning contingent size.** The Statistical characteristics of the EXACT strategy’s dismissal ratios refined by planning contingent size are depicted by a boxplot.

Figure 7 portrays in a boxplot the statistical aspects of the EXACT strategy with respect to the size of the planning contingent. The boxplot shows an adjusted mean value indicated as a blue dot, besides the mean value highlighted in red. The adjusted mean value represents the mean dismissal ratio with respect to the planning contingent size disregarding dismissals resulting from a penalty timeout of the mathematical program. The computational time limit of the EXACT approach was set at 300 seconds. The boxplot reveals an increase in the proportion of dismissals resulting from the penalty timeout with a growing size of the planning contingent. For groups of sizes between 81 and 110 patients, 11.63% of the dismissals were due to the penalty timeout. This phenomenon may be explained by considering the complexity of the mathematical program: 

•An admission and assignment is computed for a maximum of 110 patients at once.

•The virtual hospital environment contains 43 wards in total.

•The maximal planning time frame is limited to 40 days.

•Thus, the size of the mathematical program is up to: 

− 110 · 43 · 40 = 189,200 decision variables,

− 1 + 110 + 43 · 40 = 1831 constraints.

**Figure 8:**
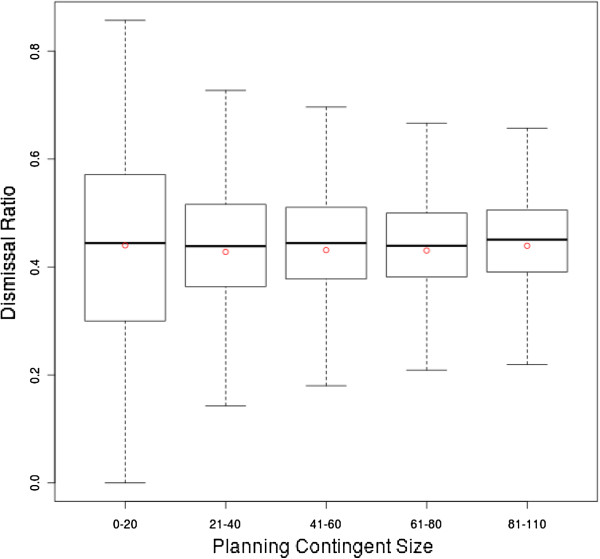
**Boxplot of the RAND strategy’s dismissal ratios refined by planning contingent size.** The statistical characteristics of the RAND strategy’s dismissal ratios refined by planning contingent size are depicted by a boxplot.

Although the number of variables and constraints does not by itself allow of reasoning about the computational complexity in general, MIPs with these characteristics are usually considered hard to solve. The boxplot portrayed in Figure 7 shows that the dismissal ratio realized by the EXACT strategy, neglecting dismissals from the penalty timeout, has a maximum which is 0.4119 lower than those of the heuristic strategies with ca. 0.43 as portrayed in Figure 8.

Figure 8 shows the dismissal ratio with respect to the planning contingent size of the RAND strategy in a boxplot. The statistical characteristics (mean ca. 0.43, median ca. 0.44, first quartile ca. 0.38, third quartile ca. 0.51) of this aspect are almost the same as those of the heuristic strategies and are thus only portrayed for the RAND strategy and not for the LEPT and SEPT strategies. The boxplot reveals that the planning contingent size has no influence on the dismissal ratio. Interestingly, the interquartile range is greater for planning contingent classes of up to 20 patients (ca. 0.27) compared to all other classes (ca. 0.14) in both the EXACT and the heuristic strategies.

#### Performance

**Figure 9:**
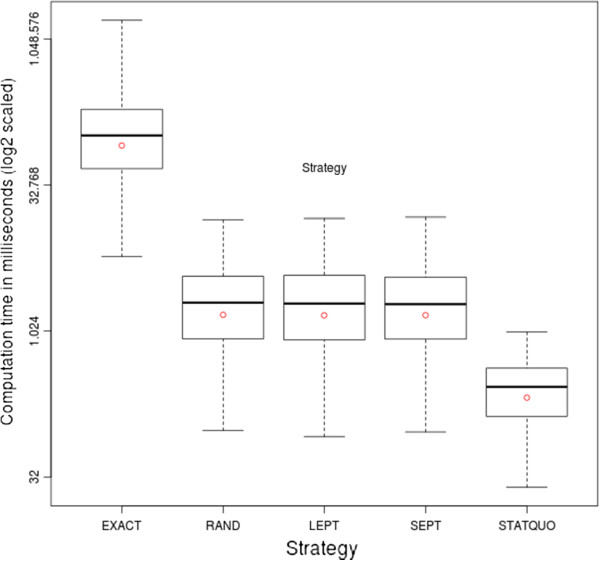
**Boxplot of computation time with respect to strategy.** The statistical characteristics of the computation time of an assignment, differentiated by strategy, are highlighted by a boxplot. The y-axis is logarithmically scaled (base two).

Figure 9 depicts the statistical aspects of the computation time for the different strategies in a boxplot (the y-axis is base two logarithmically scaled). The boxplot reveals a drastically longer computation time needed by the EXACT strategy compared to the heuristic ones. The EXACT strategy is characterized by a mean value of ca. 141 seconds and a median of ca. 106 seconds, whereas the heuristic strategies are characterized by a mean value of ca. 2.6 seconds and median value of ca. 2 seconds, thus the EXACT strategy takes almost fifty times longer than the heuristic ones.

Furthermore, the boxplot shows a strong similarity in the statistical aspects (first quartile ca. 842, mean ca. 2589, median ca. 2006, third quartile ca. 3739) between the heuristic strategies, which indicates that the sorting overhead can be neglected.

#### Cost factors

**Figure 10:**
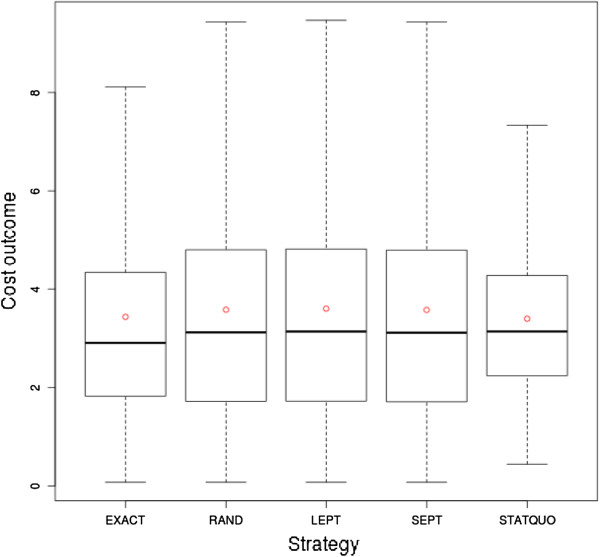
**Boxplot of total cost comparison of strategies.** The boxplot shows the statistical characteristics of the total cost outcome refined by strategy.

Figure 10 shows statistical aspects of the total cost outcome of the planning strategies in a boxplot. Dismissals were not considered in the statistical analysis of the cost factors, since the dismissal penalties would bias the cost outcome drastically. The boxplot indicates a slightly lower mean and median realization for the EXACT strategy (mean ca. 3.4338, median ca. 2.9049) compared to the heuristic ones (mean ca. 3.58, median ca. 3.12). Furthermore, the boxplot reveals similar statistical aspects between the heuristic strategies (mean ca. 3.58, median ca. 3.12, first quartile ca. 1.72, third quartile ca. 4.80).

**Figure 11:**
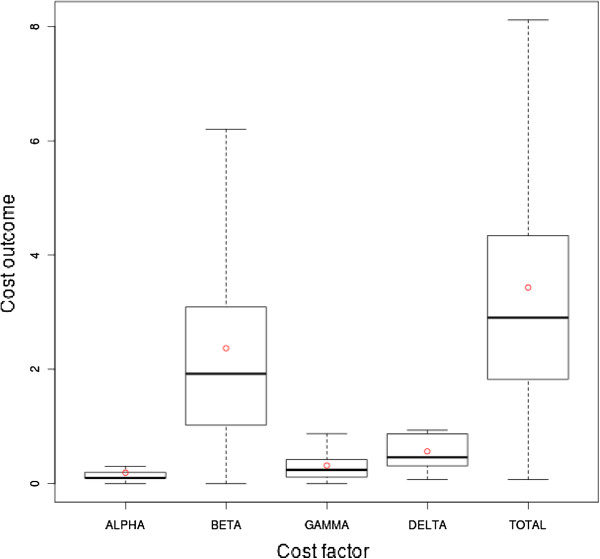
**Boxplot of the EXACT strategy’s cost factors.** The boxplot shows the statistical characteristics of the EXACT strategy refined by cost factor.

Figure 11 shows statistical aspects of the cost factor contribution of the EXACT strategy in a boxplot. The boxplot shows that the *β* cost factor (mean ca. 2.369, median ca. 1.92, first quartile ca. 1.019, third quartile ca. 3.093) is the most influential cost factor on the total outcome. The second most influential factor is the *δ* cost factor (mean ca. 0.56, median ca. 0.456, first quartile ca. 0.305, third quartile ca. 0.869).

**Figure 12:**
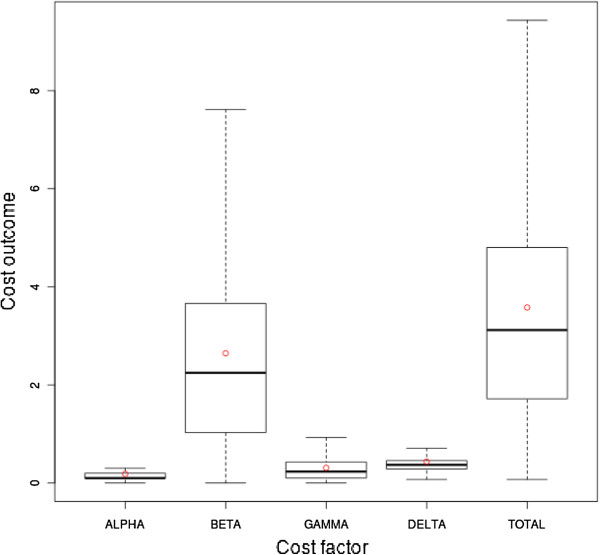
**Boxplot of the RAND strategy’s cost factors.** The boxplot shows the statistical characteristics of the RAND strategy refined by cost factor.

Figure 12 shows statistical aspects of the cost factor contribution of the RAND strategy in a boxplot. The statistical characteristics with respect to the partial cost factors were very similar between the heuristic strategies. Hence, only the statistical characteristics of the RAND strategy are analyzed further. The boxplot shows that the *β* cost factor (mean ca. 2.645, median ca. 2.247, first quartile ca. 1.027, third quartile ca. 3.662) is the most influential cost factor on the total outcome as well.

Regarding the mean value, the contribution of the *β* cost factor to the overall costs was approximately 74% when using heuristic strategies and 69% in the case of the EXACT approach. Accordingly, the contribution of the *δ* cost factor was 12% for the heuristic strategies and 16% for the EXACT approach. Thus, considering the EXACT strategy, the *δ* cost factor contributes slightly more to the total cost, while the *β* cost factor contributes less, in contrast to the heuristic strategies. The overall lower cost of the EXACT approach was thus accompanied by a reduction of the *β* and an increase of the *δ* cost factor, compared to the heuristic strategies. A higher contribution of the *δ* cost factor is attributed to a wider use of the planning time frame. Hence, the planning time frame is used more broadly by the EXACT strategy compared to the heuristic one and results in an overall lower cost outcome and a lower dismissal ratio.

## Discussion

Prior work, and the interviews conducted for the elicitation of the requirements, showed the great potential of computer aided decision support (CDS) applied to patient admission planning and assignment. CDS has been shown to improve the hospital’s resource utilization. In general, all the resources necessary for the treatment of each patient must be considered during the planning process. In contrast, our work focuses on a single resource: the bed capacity. Other important and critical resources, such as the hospital staff or the operating theater, and their interdependencies, were neglected. Nonetheless, the case managers must consider these resources during their planning task as well. Therefore, the improvements achieved by the optimization approaches may not be reproduced at the same level in real life. However, bed capacity is one of the most important resources and considerably affects decisions in patient admission planning. The availability of intensive care beds has been reported to be a major bottleneck at UK Aachen, having a direct influence on the cancellation of surgeries. Our decision support approach, based on up to date LoS estimates, is likely to be more accurate than the static approach which uses fixed LoS estimates, since changes of resource use are taken into account by the algorithmic planning methodology immediately. Our approach closely follows – often unpredictable – changes of the real situation and can thus be assumed to trigger more accurate decisions

Using individual estimates of LoS has specific advantages and drawbacks: on the one hand, changes in the LoS can be considered immediately in planning, and may reflect the complex individual conditions of patients. On the other hand, the individual estimates may be inaccurate and depend on the staffs’ experience and training. Chow et al. proposed an alternative, by deriving scheduling guidelines from recurring patterns of optimized schedules generated by their simulations: the guidelines are designed to improve the scheduling decisions without implementing optimization algorithms [35]. Demeester describes a patient admission scheduling algorithm considering hospital beds and providing operational decision support concerning the hospital bed assignment task as well [36]. In contrast to our approach, Demeester assumed that the patient’s admission date and expected LoS are known in advance [36]. Furthermore, adaptations of the patients’ LoS cannot be carried out, and hence are not considered by the decision support method. The consolidated approach proposed by our work may yield an improved robustness in the reliability of the capacity estimates. Furthermore, our approach can be generalized by considering universal resource capacities by future investigations. The evaluation of our approach reveals its principal qualification. Further investigations should study the potential of this developed DSS in clinical practice.

The limitation to a single resource, i.e., hospital beds, could be relaxed by considering the patients’ clinical pathways. Following this approach might further improve this method of decision support [43,44]. However, the consideration of individual clinical pathways will probably increase the planning complexity and uncertainty. Furthermore, clinical pathways are neither comprehensively standardized nor clinically well established.

In addition, the existence of emergency patients which have to be treated immediately heavily influences the planning problem. Emergency patients arrive at random and cannot be scheduled in advance. A common approach to considering emergency patients in advance is to provide a contingent of extra beds. However, the provision of extra beds causes further expenses for the hospital. The fixing of an extra bed contingent size is a strategic decision to be made by the hospital’s management. Decisions on the choice of resource capacity can be supported by queuing theory approaches [15], in combination with a simulation and flow analysis [45,46], or by stochastic processes [47] in general. All these approaches require accurate and detailed annual statistics of all the relevant planning aspects of emergency patients. The proposed DSS does not take into account the occurrence of emergency patients, but assumes a sufficient amount of extra beds reserved for emergency patients. However, an explicit consideration of emergency patients by the DSS might further improve this decision support method as well.

The transfer of patients to other beds is considered only implicitly. Due to ward abstraction, i.e., bed allocation irrespective of individual beds, transfers within the same wards can be ignored. Transfers to different wards may be interpreted as a discharge from the prior ward and an assignment to the subsequent ward. Following this interpretation, transfers are implicitly regarded by the DSS. However, taking transfers explicitly into account in advance might also further improve the DSS.

Overall, the DSS might be improved by considering further critical resources, the clinical pathway of patients, emergency patients, algorithmic improvements, and a weight adaptation of the cost factors.

### Requirements analysis

The interviewees reported a classification of patients into priority groups reflecting their treatment urgency. The characteristics of the patients in the different priority groups were not further analyzed. However, an analysis of the priority groups might reveal interesting patterns which could be used to improve patient admission planning as well. For instance, it might happen that certain clusters of patients appear in the groups, perhaps leading to the exhaustion of certain resources. Besides, further analysis of the characteristics of the priority groups might be interesting in order to adapt the resource availability to reflect upcoming demand and to avoid congestion.

### Comparison between the heuristic approaches and the exact solution

Given the similarity of all the heuristic approaches in their performance, we argue that it is sufficient to compare the performance of only one heuristic approach with the exact solution. The complexity of the heuristic algorithms (reported in the methods section) justifies this argument: considering a maximum of 110 patients to be scheduled, the sorting complexity may well be neglected. The comparison of the dismissal ratios is complicated by the fact that there are two genuinely different types of dismissals in the case of the EXACT approach: 1) dismissals due to exhausted bed capacity 2) dismissals due to exceeding the time limit of the algorithm. The first kind of dismissal should clearly be minimized by the exact solution of the optimization problem. Considering the effect of the timeout, the strong positive correlation between the size of the group to be scheduled and the dismissal ratio in the EXACT case is not surprising.

### Comparison with the baseline STATQUO approach

The baseline approach used in our study only pays regard to a subset of the aspects already considered by the optimization approach. In a real life scenario, further aspects, such as the operating room capacities and other critical resource capacities, are usually taken into account by the persons in charge. Therefore, the STATQUO criteria might not always guide real life decisions.

### Weight adaptation of the cost factors

All weight factors of the partial cost factors were fixed to 1.0. Hence, each cost factor is regarded as having equal weight. The weight factors can serve to adjust the impact of the partial cost factors, depending on the aim of the optimization strategy. A suitable weight combination for a given strategy needs to be investigated by an additional study.

### Algorithm improvements

Although our approach allows of adaptable LoS estimates and ward utilization estimates, stochastic variability is neglected in the final BIP. To enable an exact solution within an acceptable time, the stochastic mathematical program was reduced to a deterministic BIP. A stochastic mathematical program might lead to an improved assignment, due to a more accurate representation of the modeling reality. Discretization of a stochastic program is usually performed by substituting the random variables by constant values. The constant values are usually obtained by adding some slack value, e.g. the standard deviation, to a constant factor, e.g. the mean outcome of the substituted random variable. Thus, the resulting program does not represent the reality as accurate as the stochastic program, due to neglecting the stochastic variability. However, an exact stochastic mathematical program of the necessary complexity will most likely fail to solve the problem within an acceptable time [48].

The bed assignments calculated by the heuristic strategies might be improved by applying meta-heuristic strategies, such as Tabu Search [36], Evolutionary Approaches [49], or Simulated Annealing [29,50].

### Simulation procedure

The simulation conducted to evaluate the model used data taken from a real hospital setting. The main characteristics and quantities of the model, namely the capacity and configuration of the wards, the linkage between the wards and the clinics, and the distribution of the treating clinics, correspond to the situation at UK Aachen. Besides, the patients’ characteristics were based on the real distributions as well. The distributions of the planning collective size, gender, treatment priority, and individual patient preferences were derived from the interviews. The statistics concerning the number and type of treatments refer to the annual report of UK Aachen [42]. The LoS distribution was given by the German national DRG statistics as provided by the DRG-Browser. In this way, errors resulting from unrealistic or biased simulation data could be minimized. However, the simulation probably still possesses unrealistic patient datasets, for instance, male gynecology patients.

The simulation revealed a quite high dismissal ratio, of approximately 40% realized by the EXACT and heuristic strategies, and approximately 74% realized by the STATQUO approach. This high ratio may be attributed to the fact that each patient is considered exactly once for admission planning. Patients who cannot be planned for at the planning time point are dismissed and will not be planned for in the future. In reality, a patient that cannot be planned for at the planning time point will usually be considered for planning again during the patient’s planning period, and is thus not considered as dismissed at that point in time. A dismissal ratio of 40% may be interpreted as meaning that 60% of the patients on the list are planned successfully, whereas 40% could not be assigned.

The effect of LoS revision was not analyzed within the simulation study due to the complexity of such an analysis. In contrast to the LoS estimations (which are based on the DRG statistics), a reliable data source for a realistic simulation of LoS adaptions was not available. Thus, we had to postpone a simulation based evaluation of the effects of LoS adaptations. In the future, the necessary data can be systematically acquired during the routine use of the module proposed in this paper; the organizational prerequisites for the routine use have yet to be provided. Nonetheless, we could show the feasibility of using a cost-based patient admission assignment methodology, which takes into account adaptable length of stay estimations and aggregated resources. Precisely analyzing LoS adaption effects clearly is an important and valuable topic for ongoing research.

## Conclusion

Our requirements analysis revealed a strong need for computer-based decision support in the context of case and bed management.

Our work focused on the implementation of a decision support system for admission planning and bed assignment, taking into account the availability of suitable hospital beds. Decision support relies on an algorithmic core, providing the calculation of an optimal admission and assignment plan for a given group of patients and its implementation within a software system.

Patient admission and assignment is based on up to date and adaptable LoS estimates, taking into account the aggregated contingents of hospital beds, treatment priorities, patient preferences, and a linkage between clinics and wards.

The admission planning and assignment problem was formally described by a BIP. In order to solve the BIP, two classes of strategies were developed and analyzed: the first class consisted of an exact approach, and the other class contained three heuristic strategies. Four partial cost factors have been introduced in order to represent the advantage of an assignment: affinity costs, ward occupancy, change of ward occupancy, and assignment delay. The weighted sum of the partial cost factors results in the assignment costs. The objective of the BIP is to minimize the total assignment costs and is, thus, following a min-cost strategy.

Discrete event simulation revealed the following facts: the application of optimization strategies following a min-cost approach yields a marked reduction in patient dismissals compared to a status-quo approach. In theory, this results in an increase in ward utilization. In addition, calculating the exact solution with a MIP solver resulted in only minor advantages with respect to costs and the dismissal ratio compared to the heuristic strategies. Moreover, calculating an adequate exact solution requires almost fifty times more computational time than the heuristic strategies. Our study analyzed and compared three heuristic strategies: the LEPT, SEPT, and RAND strategies. LEPT and SEPT did not reveal considerable advantages over the RAND strategy, and should thus be omitted because of the computational load resulting from the sorting procedure. An adequate solution to the bed assignment problem is calculated much faster by the heuristic strategies than by the exact optimization. Thus, the RAND strategy utilizing a min-cost optimization approach can be considered as the preferred method for bed assignment in the case of shared resources.

The simulation revealed a promising reduction in the patient dismissal rate by applying the proposed DSS strategies and, hence, may present a way to increase the hospital’s throughput in the future.

## Endnote

^a^http://scip.zib.de/^b^http://plato.asu.edu/ftp/milpc.html^c^http://www.g-drg.de^d^http://www.g-drg.de

## Abbreviations

BIP: Binary Integer Program; CM: Case Management; DRG: Diagnosis Related Group; DSS: Decision Support System; EXACT: Exact strategy of solving a BIP; LEPT: Longest Expected Processing Time; LoS: Length of Stay; MIP: Mixed Integer Program; RAND: Random assignment strategy; SC: Standard Care; SEPT: Shortest Expected Processing Time; STATQUO: Status quo assignment strategy

## Competing interests

The authors declare that they have no competing interests.

## Authors’ contributions

All authors contributed to the interviews of the requirements elicitation, the design of the mathematical model, and the conceptual design of the decision support system. RS prepared, conducted, and evaluated the interviews, developed and implemented the mathematical model resulting in the decision support system, and designed and performed the evaluation. SG and CS were involved in the design and the evaluation of the interviews, the design of the mathematical model, of the decision support system, and its evaluation. All three authors have read and approved the final manuscript.

## Pre-publication history

The pre-publication history for this paper can be accessed here:

http://www.biomedcentral.com/1472-6947/13/3/prepub
